# Failure to Detect Mutations in *U2AF1* due to Changes in the GRCh38 Reference Sequence

**DOI:** 10.1016/j.jmoldx.2021.10.013

**Published:** 2022-03

**Authors:** Christopher A. Miller, Jason R. Walker, Travis L. Jensen, William F. Hooper, Robert S. Fulton, Jeffrey S. Painter, Mikkael A. Sekeres, Timothy J. Ley, David H. Spencer, Johannes B. Goll, Matthew J. Walter

**Affiliations:** ∗ Division of Oncology, Department of Internal Medicine, Washington University School of Medicine, St. Louis, Missouri; † Siteman Cancer Center, Washington University School of Medicine, St. Louis, Missouri; ‡ McDonnell Genome Institute, Washington University School of Medicine, St. Louis, Missouri; ∗∗ Department of Pathology and Immunology, Washington University School of Medicine, St. Louis, Missouri; § The Emmes Company, Rockville, Maryland; ¶ Moffitt Cancer Center, Tampa, Florida; ‖ Division of Hematology, Department of Medicine, Sylvester Comprehensive Cancer Center, University of Miami School of Medicine, Miami, Florida

## Abstract

The *U2AF1* gene is a core part of mRNA splicing machinery and frequently contains somatic mutations that contribute to oncogenesis in myelodysplastic syndrome, acute myeloid leukemia, and other cancers. A change introduced in the GRCh38 version of the human reference build prevents detection of mutations in this gene, and others, by variant calling pipelines. This study describes the problem in detail and shows that a modified GRCh38 reference build with unchanged coordinates can be used to ameliorate the issue.

The *U2AF1* gene encodes a core component of the mRNA splicing machinery that is frequently mutated in myelodysplastic syndrome (MDS) and other cancers.^1^, ^2^, ^3^ Though predominantly associated with hematopoietic cancers (73%), mutations are also recurrent in lung tumors (6.5%) and have been reported in 24 other tumor types. Specifically, mutations at residues S34 and Q157 have been shown to promote exon skipping and are confirmed driver mutations contributing to cancer pathogenesis.^4^, ^5^, ^6^, ^7^

## Materials and Methods

Genomic data were collected as part of the MDS National History Study or The Cancer Genome Atlas (TCGA) project and appropriate consent was received under those protocols.^8^^,^^9^ Sequencing reads from the MDS cohort were aligned to both masked and unmasked GRCh38 reference genomes using the BWA-MEM software package version 0.7.15 (*https://github.com/lh3/bwa/releases/tag/v0.7.15*),^10^ followed by sorting and deduplication, as detailed in a CWL (Common Workflow Language) workflow archived at *https://git.io/JYbGl* (last accessed December 1, 2021). Variants in the MDS cohort were called in single-sample mode using VarScan software version 2.4.2 with params “--min-coverage 20 --min-reads2 5 --min-var-freq 0.05 --strand-filter 1”.^11^ Data from TCGA acute myeloid leukemia samples were aligned using the same process, and somatic variants were called using an ensemble approach, described in detail in the CWL workflow at *https://git.io/JYbGM* (GitHub, last accessed December 1, 2021). The modified genome FASTA file used for these analyses is available at *https://zenodo.org/record/4684553* (Zenodo, last accessed December 1, 2021).

Sequence data from the MDS cohort are available in the database of Genotypes and Phenotypes (dbGaP) under accession id phs002714.v1.p1. The study is currently available at *https://www.ncbi.nlm.nih.gov/projects/gap/cgi-bin/study.cgi?study_id=phs002714.v1.p1* (dbGaP, last accessed January 23, 2022). TCGA acute myeloid leukemia data are available via the Genomic Data Commons at *https://portal.gdc.cancer.gov* (last accessed December 1, 2021). Pipelines used for variant calling are available at *https://github.com/genome/analysis-workflows* (GitHub, last accessed December 1, 2021). All links in the Materials and Methods are to the specific versions of the workflows used.

## Results

**Figure 1:**
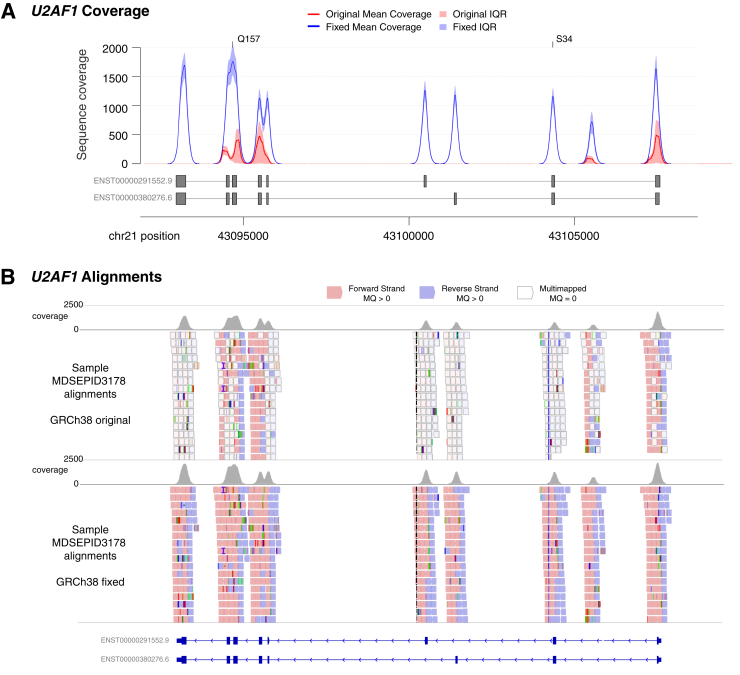
Alignment issues across the U2AF1 locus. **A:** Sequence coverage of reads with mapping quality (MQ) >0 across the *U2AF1* gene for 120 bone marrow samples sequenced after capture with a custom reagent. The mean coverage for realignments to GRCh38 are shown in red, whereas the mean coverage for alignments to the authors’ modified GRCh38 reference is shown in blue. Shading indicates the interquartile range (IQR) for each. Exons from the two primary protein-coding isoforms are shown below, and the locations of hotspot mutations at amino acids S34 and Q157 are indicated. **B:** An Integrated Genomics Viewer (IGV) view of sequence reads, with alignments to GRCh38 at top and alignments to the modified reference at bottom. Grey bars at top show overall coverage. Reads in white indicate multimapped reads, with mapping qualities of zero, whereas red and blue reads have higher quality alignments.

As part of the National Myelodysplastic Syndrome (MDS) Natural History Study (ClincialTrials.gov, *https://www.clinicaltrials.gov*, identifier: NCT02775383), a targeted gene panel was used to sequence bone marrow samples from 120 patients either diagnosed with MDS or suspected to have MDS.^8^ Of these patients, 38 were eventually confirmed to have MDS or a myeloproliferative neoplasm. Initial analyses looking at sequencing quality metrics revealed coverage levels and mutation frequencies that closely matched expectations, with one exception: mutations in the *U2AF1* spliceosome gene are typically observed in nearly 10% of MDS or myeloproliferative neoplasm patients, but only 2 of the 38 MDS or myeloproliferative neoplasm patients (5.2%) in this group had such mutations, both at the Q157 hotspot.^1^^,^^12^^,^^13^ Although this deviation was not significant (compared with the Walter et al^12^ cohort; *P* = 0.53 via Fisher's exact test), both mutations had only about 15% of the expected sequence coverage (mean of 204× depth), whereas the full targeted panel had a median coverage of 1337.3× (Supplemental Table S1). Nearly the entirety of the *U2AF1* gene was likewise affected, with a median depth of only 130× and four exons with no coverage at all (Figure 1). Manual inspection of the alignments with Integrated Genomics Viewer (IGV) revealed that this poor coverage extended to a 150-kb region of the genome where the majority of reads had a mapping quality of zero (Figure 1).^14^ The sequence aligner BWA-MEM reserves this mapping quality zero score for reads that cannot be uniquely placed in the genome, and it generally indicates a highly repetitive sequence or problems with the underlying assemblies.^10^ The region with such problematic alignments spans the *CBS*, *U2AF1*, *FRGCA*, and *CRYAA* genes.

Further investigation revealed that in the GRCh38 reference build, content added to the p-arm of chromosome 21 (chr21:6427259-6580,181) contained sequence that replicated the sequence of the *U2AF1* locus (chr21:43035875-43187577) with 99.0% identity. The same issue does not exist in prior reference builds GRCh36 or GRCh37. After consultation with members of the Genome Reference Consortium (GRC), it was determined that a bacterial artificial chromosome (BAC) clone (Nucleotide, *https://www.ncbi.nlm.nih.gov/nuccore*; accession number FP236240.8) was incorrectly added to the reference genome, creating this duplicate sequence. This resulted in the alignment algorithm, BWA-MEM, splitting the reads among these two loci, thus lowering the overall coverage substantially (Figure 1, Supplemental Figure S1, and Supplemental Table S1). In addition, reads with mapping quality scores of zero are typically excluded or down-weighted during variant calling, due to the increased chance of artefactual calls, and these factors combined explained the paucity of mutations observed in *U2AF1*, especially at the S34F position.

To address this issue, the authors’ group created a modified version of GRCh38 that maintains the coordinate system, but masks the new duplicate sequence on chromosome 21p by replacing it with “N” characters. The authors realigned the data to this reference and observed a substantial increase in coverage and mapping qualities across the affected region. Over the exons of *U2AF1*, the coverage of reads with mapping quality >0 rose from a median of 0.3× to a median of 1195×. This enabled the discovery of an additional *U2AF1* mutation (S34F) in this MDS cohort. The data were also aligned to GRCh37, and it was confirmed that this region was not problematic in the older reference, where the median coverage over the exons of *U2AF1* was 1381× (Supplemental Table S1 and Supplemental Figure S2).

To validate this finding in an orthogonal data set, data were retrieved from acute myeloid leukemia patients sequenced for the TCGA paper.^9^ The data in this study were originally aligned to genome build GRCh36, and six mutations in the *U2AF1* gene were reported from exome sequencing. After this paper was published, the Genomic Data Commons took this sequence dataset, realigned it (with BWA-MEM) to the stock GRCh38.d1.vd1 reference, and produced variant calls using four different algorithms.^10^^,^^15^ These variant files [in MAF (Mutation Annotation Format)] reported no *U2AF1* mutations in the six expected samples. Their GRCh38 sequence alignments were then downloaded, and the same coverage and mapping quality issues on chromosome 21 were observed. One sample lacked any *U2AF1* reads at all, due to unrelated sequencing problems (the TCGA consortium only identified the mutation using orthogonal assays). This sample was excluded from further analyses, leaving five evaluable samples.

Running another somatic variant calling pipeline on these alignments also did not reveal any *U2AF1* mutations. However, after realigning the sequence data to the masked version of GRCh38, the same somatic pipeline identified all five expected mutations in *U2AF1* (Supplemental Table S2).

## Discussion

The reference genome is essential to modern cancer genomics, but problems with these assemblies have the potential to cause both false positive and negative results. In this study, the authors describe the latter, where changes introduced in the GRCh38 human reference build cause mutations in a cancer driver gene to be missed with standard analysis approaches. GRCh38 was first released in late 2013, but widespread adoption lagged somewhat, so some studies involving myeloid malignancies (TCGA, the Beat AML trial) have been spared this issue because they used older versions of the reference.^9^^,^^16^ Nonetheless, large cancer databases, including the National Cancer Institute's Genomic Data Commons, are likely missing many *U2AF1* mutations due to this artefact. This also has clinical implications because *U2AF1* mutations have strong associations with prognosis, and clinical trials of splice-modulating drugs are being planned or are underway.^5^^,^^17^^,^^18^

These findings have been reported to the GRC (*https://www.ncbi.nlm.nih.gov/grc/human/issues/HG-2544*, last accessed December 10, 2021), but as there are no patches or new releases scheduled for the human reference genome, the problem remains unresolved in the current release of GRCh38 (GRCh38.p13). However, in the time since these analyses were performed, the GRC has released a masking file that includes this chr21 region, along with two other contaminating sequences on alternate contigs.^19^ Using this file to create a masked genome mirrors the approach that the authors’ analyses used and likewise resolves the issues in *U2AF1* reported in this paper.

To apply this fix, the bed file can be downloaded from NCBI at *https://ftp.ncbi.nlm.nih.gov/genomes/all/GCA/000/001/405/GCA_000001405.15_GRCh38/seqs_for_alignment_pipelines.ucsc_ids/GCA_000001405.15_GRCh38_GRC_exclusions.bed* (last accessed December 1, 2021), then applied to a GRCh38 FASTA file using the bedtools maskfasta tool, followed by reindexing with the aligner of choice.^20^

Although masking the genome offers better quality alignments at this locus, the next leap forward will come with a new reference sequence, likely based on the draft genome recently produced by the Telomere-to-Telomere consortium.^21^ In the longer term, the genomics community can look forward to graph genome approaches capable of representing haplotypes from many different populations. These should further increase the accuracy of short-read genomic alignments, upon which many analyses, and increasingly clinical decisions, are based.

As these genome reference improvements are released, the genomics community will need to validate them before they can be used on clinical cases. Previous data sets will need to be realigned to ensure that changes are understood and problematic portions of assemblies that might alter diagnostic results are identified. Genomic annotations and pipelines will also need to be updated, which can be resource intensive. Hence, GRCh38 will likely remain in use for years. The use of GRC-released bed file is recommended to create a masked reference that is coordinate-compatible to be used interchangeably with the standard GRCh38 reference in cancer genomics applications. Especially, applications where detection of *U2AF1* mutations is critical, including sequencing of hematological cancers or studies of spliceosome dysfunction.

## Supplemental Data

Supplemental Figure S1 Examples of poor coverage of other genes in the affected region. Examples shown are from exome sequencing of sample TCGA-AB-2912-11A. Integrated Genomics Viewer (IGV) views show sequence reads, with alignments to GRCh38 at **top** and alignments to the modified reference at bottom. Grey bars at top show overall coverage. Reads in white indicate multimapped reads, with mapping qualities of zero, whereas red and blue reads have higher quality alignments. **A:** Reads aligning to the *CBS* gene. **B:** Reads aligning to the *CRYAA* gene (*FRGCA*, the other gene in the affected region, was not targeted by this exome reagent).

Supplemental Figure S2 Myelodysplastic syndrome (MDS) data aligned to GRCh37 from sample MDSEPID3178 (the same sample shown in Figure 1), showing no evidence of alignment issues. The *U2AF1* transcript shown is ENST00000291552.9 (Ensembl, *ensembl.org/index.html*, last accessed January 10, 2022).
