# Supplementary figures and images for: Failure to Detect Mutations in U2AF1 due to Changes in the GRCh38 Reference Sequence
Source: J Mol Diagn. 2022 Mar;24(3):219–23. doi: 10.1016/j.jmoldx.2021.10.013 (PMC8950341; doi:10.1016/j.jmoldx.2021.10.013)

A

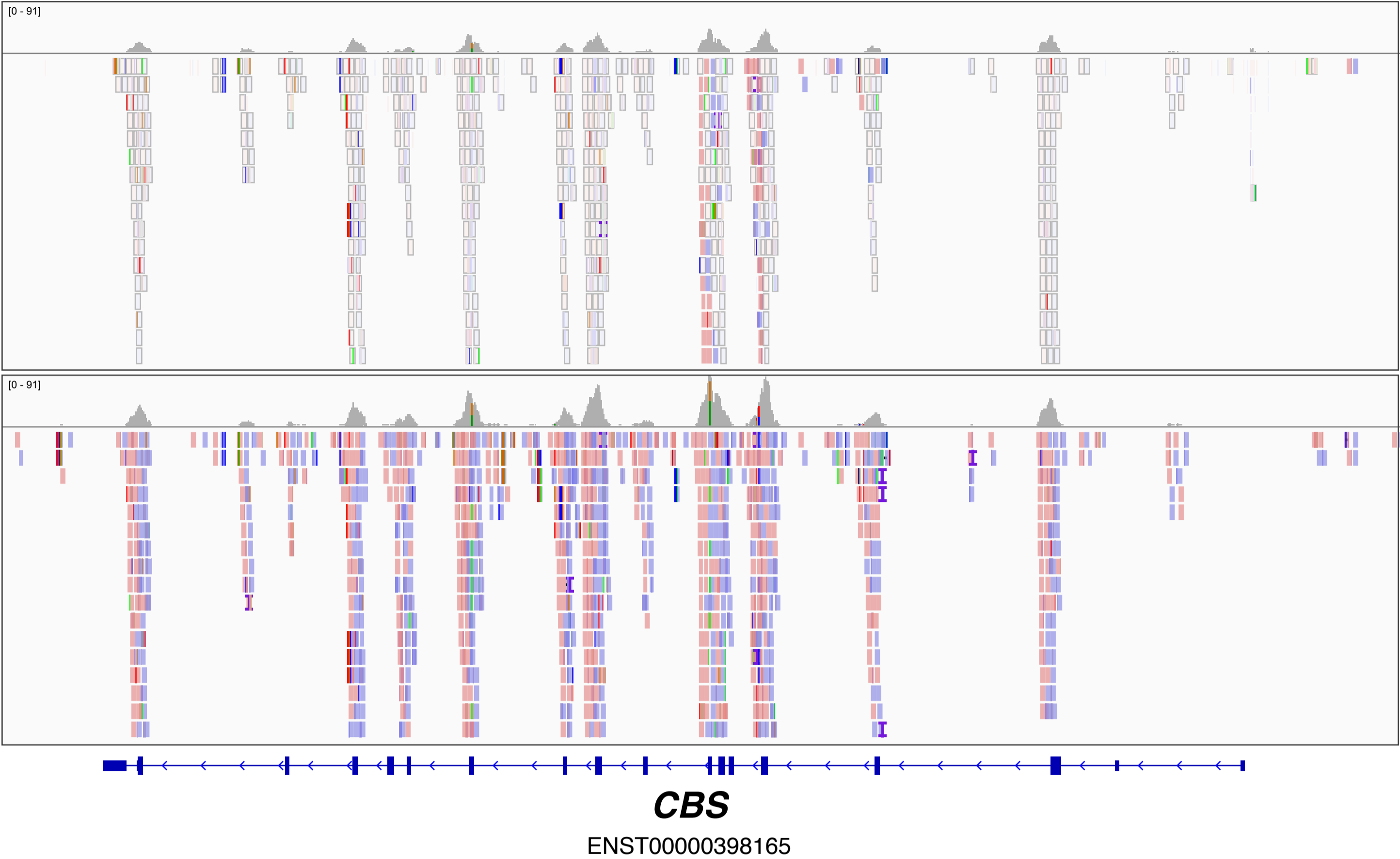

B

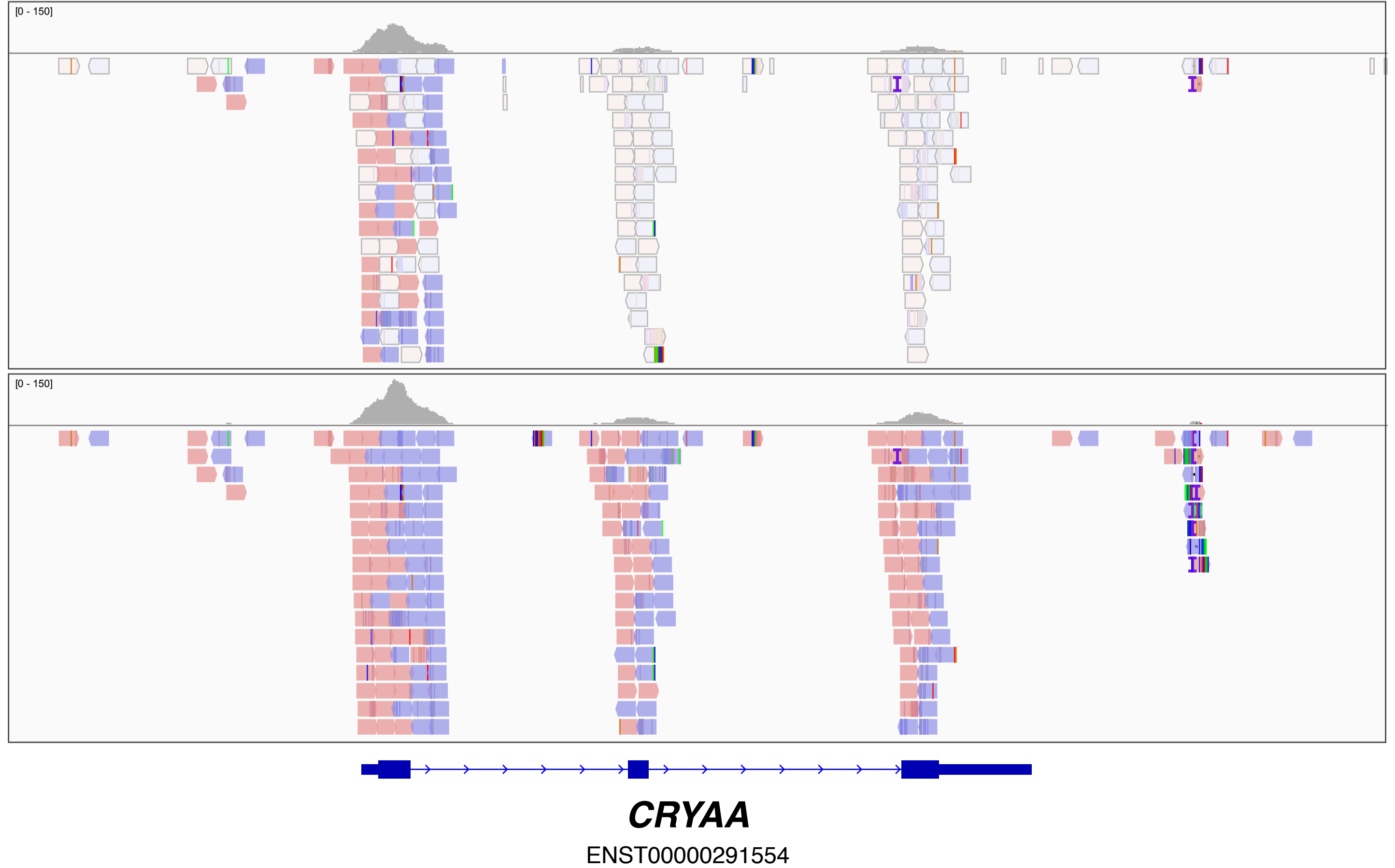

Supplement: Supplemental Figure S1 — Examples of poor coverage of other genes in the affected region. Examples shown are from exome sequencing of sample TCGA-AB-2912-11A. Integrated Genomics Viewer (IGV) views show sequence reads, with alignments to GRCh38 at top and alignments to the modified reference at bottom. Grey bars at top show overall coverage. Reads in white indicate multimapped reads, with mapping qualities of zero, whereas red and blue reads have higher quality alignments. A: Reads aligning to the CBS gene. B: Reads aligning to the CRYAA gene (FRGCA, the other gene in the affected region, was not targeted by this exome reagent). [file mmc1.pdf]

[0 - 4383]

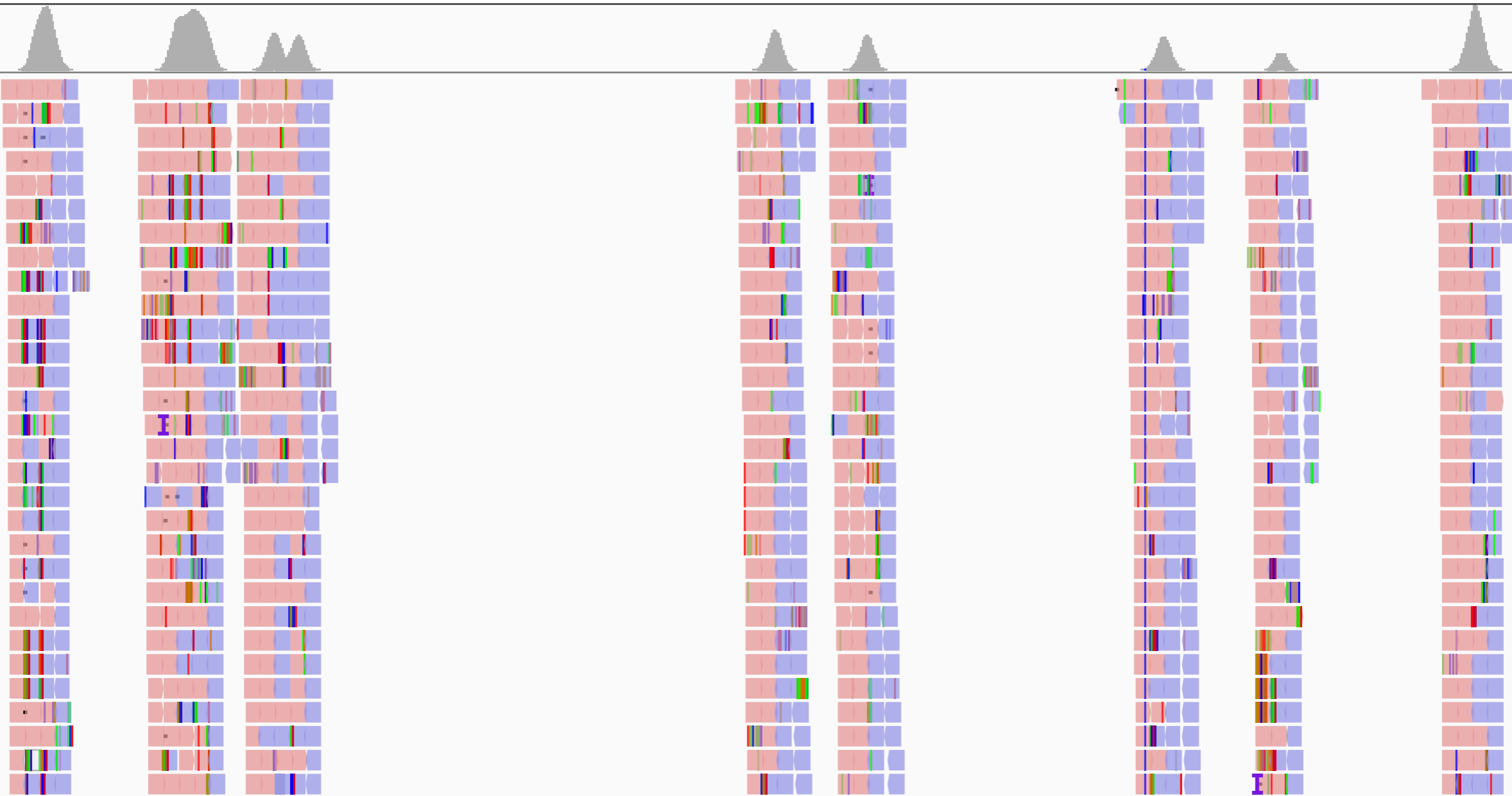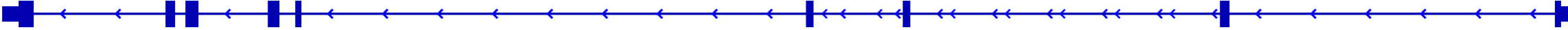

***U2AF1***

ENST00000291552

Supplement: Supplemental Figure S2 — Myelodysplastic syndrome (MDS) data aligned to GRCh37 from sample MDSEPID3178 (the same sample shown in Figure 1), showing no evidence of alignment issues. The U2AF1 transcript shown is ENST00000291552.9 (Ensembl, ensembl.org/index.html, last accessed January 10, 2022). [file mmc2.pdf]
