# Supplementary material for: Failure to Detect Mutations in U2AF1 due to Changes in the GRCh38 Reference Sequence
Source: J Mol Diagn. 2022 Mar;24(3):219–23. doi: 10.1016/j.jmoldx.2021.10.013 (PMC8950341; doi:10.1016/j.jmoldx.2021.10.013)
Supplement: Supplemental Table S2 [file mmc4.docx]

**Supplemental Table S2:** Variants in *U2AF1* detected from The Cancer Genome Atlas AML sample data aligned to a masked GRCh38 reference. No *U2AF1* mutations were found in the same data aligned to the unmasked GRCh38 reference.

| case | chr | pos | ref | var | gene | transcript | HGVSp | effect |
| --- | --- | --- | --- | --- | --- | --- | --- | --- |
| TCGA-AB-2912 | chr21 | 43104346 | G | A | *U2AF1* | ENSG00000160201 | ENSP00000291552.4:p.Ser34Phe | missense_variant |
| TCGA-AB-2861 | chr21 | 43104346 | G | T | *U2AF1* | ENSG00000160201 | ENSP00000291552.4:p.Ser34Tyr | missense_variant |
| TCGA-AB-2821 | chr21 | 43094667 | T | G | *U2AF1* | ENSG00000160201 | ENSP00000291552.4:p.Gln157Pro | missense_variant |
| TCGA-AB-2847 | chr21 | 43104346 | G | A | *U2AF1* | ENSG00000160201 | ENSP00000291552.4:p.Ser34Phe | missense_variant |
| TCGA-AB-2843 | chr21 | 43104346 | G | A | *U2AF1* | ENSG00000160201 | ENSP00000291552.4:p.Ser34Phe | missense_variant |

Transcripts and annotations from Ensembl release 95
